# Personalized dosimetry enhances hepatocellular carcinoma response in Yttrium-90 resin microsphere radioembolization

**DOI:** 10.1007/s00330-026-12562-z

**Published:** 2026-04-28

**Authors:** Ettore di Gaeta, Makoto Taninokuchi Tomassoni, Francesca Calabrese, Giovanni Matassa, Annarita Savi, Carla Canevari, Patrizia Magnani, Giovanna Pepe, Stephanie Steidler, Francesca Ratti, Federica Cipriani, Margherita Rimini, Andrea Casadei-Gardini, Lorenzo Braccischi, Maria Adriana Cocozza, Lidia Strigari, Giuseppe Della Gala, Arber Golemi, Elisa Lodi Rizzini, Stefano Fanti, Matteo Cescon, Matteo Serenari, Fabio Piscaglia, Maria Cristina Morelli, Arturo Chiti, Cristina Mosconi, Francesco De Cobelli

**Affiliations:** 1 Department of Radiology, IRCCS Ospedale San Raffaele, Milan, Italy; 2 Department of Radiology, IRCCS Azienda Ospedaliero-Universitaria di Bologna, Bologna, Italy; 3 Department of Nuclear Medicine, IRCCS Ospedale San Raffaele, Milan, Italy; 4 Department of Hepatobiliary Surgery, IRCCS Ospedale San Raffaele, Milan, Italy; 5 School of Medicine, Vita-Salute San Raffaele University, Milan, Italy; 6 Department of Oncology, IRCCS Ospedale San Raffaele, Milan, Italy; 7 Department of Medical Physics, IRCCS Azienda Ospedaliero-Universitaria di Bologna, Bologna, Italy; 8 Nuclear Medicine, IRCCS Azienda Ospedaliero-Universitaria di Bologna, Bologna, Italy; 9 Radiation Oncology, IRCCS, Azienda Ospedaliero-Universitaria di Bologna, Bologna, Italy; 10 Hepatobiliary Surgery and Transplant Unit, IRCCS Azienda Ospedaliero-Universitaria di Bologna, Bologna, Italy; 11 Division of Internal Medicine, Hepatobiliary and Immunoallergic Diseases, IRCCS Azienda Ospedaliero-Universitaria di Bologna, Bologna, Italy; 12 Internal Medicine Unit for the Treatment of Severe Organ Failure, IRCCS Azienda Ospedaliero-Universitaria di Bologna, Bologna, Italy

**Keywords:** Hepatocellular carcinoma, Therapeutic embolization, Yttrium radioisotopes, Personalized medicine

## Abstract

**Objectives:**

To identify a tumor-mean absorbed dose (*D*mean) that can predict response to Yttrium-90 resin-microsphere transarterial radioembolization (TARE) in patients with hepatocellular carcinoma (HCC) and evaluate its efficacy and safety.

**Materials and methods:**

Patients with HCC eligible for TARE in two centers between January 2020 and May 2024 were retrospectively analyzed. Clinical, radiological, and procedural data were collected. Objective response rate (ORR) on lesion, complete response (CR), overall response, time-to-local progression (TLP), and time-to-progression (TTP) were evaluated on contrast-enhanced CT at 3 and 6 months according to mRECIST. The optimal *D*mean of ORR on the target lesion and of CR was identified with ROC analysis at 3-months. Fischer’s test compared ORR, Kaplan–Meier survival outcomes, and Cox regression was used for uni-and multivariable analyses.

**Results:**

Seventy-six lesions in 64 patients (mean age 71.3 ± 9.6; 54 men) were evaluated. Median follow-up was 15.0 months (IQR 8.0–24.3). Mean tumor diameter was 55.2 (± 31.8) mm. CR on target lesion at 3-months was achieved in 42 lesions. Mean TLP and OS were 27.6 ± 2.5 and 36.2 ± 2.9 months, respectively. The calculated *D*mean for ORR was 296.74 Gy (specificity 100, PPV 100%). Patients treated with equal or lower doses had a significantly shorter TLP (log-rank *p* = 0.001). *D*mean > 296.74 Gy did not increase the risk of complications. A *D*mean > 435.11 Gy predicts CR*.*

**Conclusion:**

A *D*mean threshold of 296.74 Gy provides significant therapeutic efficacy without compromising patient safety. All HCC lesions treated with > 435.11 Gy achieved CR.

**Key Points:**

***Question*** *In patients with HCC treated with 90Y-resin microsphere TARE, can an optimized personalized dose improve efficacy without compromising safety*?

***Findings*** *The calculated mean dose (Dmean), even though above guideline indications, did not yield serious adverse events whilst enhancing response to treatment*.

***Clinical relevance*** *HCC recurrence is associated with poor patient outcomes. Personalized dosimetry is best practice for tailoring radioactive doses, and the cut-off dose calculated in this cohort identifies that with the best response and yields additional data on resin microsphere doses*.

**Graphical Abstract:**

**Figure:**
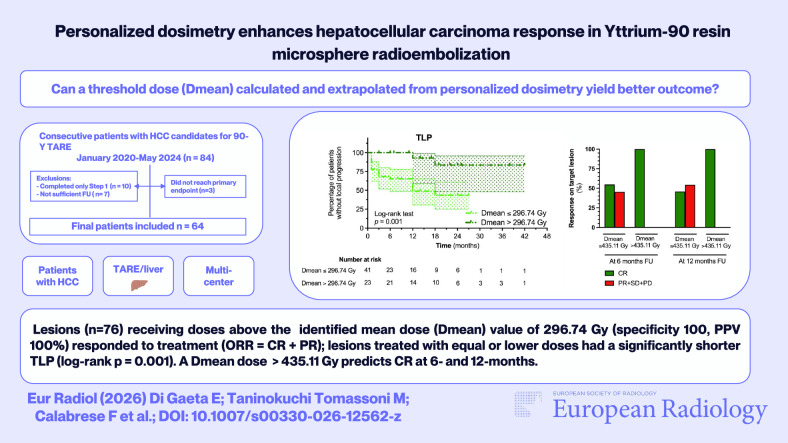

## Introduction

The non-surgical treatment of hepatocellular carcinoma (HCC) is very challenging due to the difficulty of achieving a complete response (CR), especially in intermediate and advanced stage cases, and in preserving liver function. In this scenario, the therapeutic value of transarterial radioembolization (TARE) with Yttrium-90 (^90^Y) in the management of HCC is increasing [1]. The indication of this treatment has expanded since its original conception as a bridging option for liver transplantation or palliation. Supported by the evidence of studies including the LEGACY [2] and RASER [3] studies, it was incorporated in the 2022 Barcelona Clinic Liver Cancer (BCLC) update strategy [4], as a curative treatment for patients with very early or early-stage HCC who are not suitable for surgical resection or liver transplantation. The latest European Association for the Study of the Liver (EASL) guidelines indicate this treatment for disease control in all BCLC stages [5]. The importance of personalized dosimetry in achieving optimal outcomes is well-documented, with recent literature showing improvements in both overall survival (OS) and objective response rate (ORR) [6–11]. The optimal application of TARE across various HCC stages, particularly in the context of resin microspheres and personalized dosimetry, is still challenging. Initially developed and validated for glass microspheres, personalized dosimetry protocols for resin have been published in the last quinquennium [9]. Studies have previously described the relation between dose-tumor response cut-offs and OS [12–14], with limited detail on personalized dose calculation [15].

Radiological response using modified Response Evaluation Criteria in Solid Tumors (mRECIST) [16] at approximately 3 months after TARE, has been widely adopted as the first standardized time point for objective response assessment. This time point reliably reflects early radiation-induced tumor necrosis and reduces potential misinterpretation related to transient hepatic attenuation differences (THADs) post-treatment following TARE [17–21].

The aim of this study was to evaluate the efficacy of a threshold dose (*D*mean) in patients with early to advanced-stage HCC treated with resin microspheres. The surrogate endpoint for efficacy will be ORR on the target lesion at 3 months. Safety, correlation to the different doses, OS, time-to-local progression (TLP), time-to-progression (TTP), and overall tumor response will also be evaluated.

## Materials and methods

The data collected was part of Institutional Registries approved by the local Ethics Committee of each of the two hospitals (IRCCS San Raffaele approval 64/INT/2021; IRCCS Azienda Ospedaliero-Universitaria Bologna approval number: EM109/2924_193/2021/Oss/AOUBo). Participants signed a specific written informed consent, and the study adhered to the ethical and quality standards set by the Declaration of Helsinki and the ICH Good Clinical Practice Guidelines.

This retrospective observational study on prospectively collected data at the two Institutions, included 64 patients diagnosed with HCC who underwent TARE with ^90^Y resin microspheres (SIR-spheres® Y-90 resin microspheres, Sirtex Medical Europe GmbH).

### Patients

Patients with HCC diagnosed either radiologically or pathologically and eligible for TARE were enrolled between January 2020 and May 2024, following the indication of the dedicated Institutional multidisciplinary tumor boards. Treatment methods (^90^Y-resin microspheres), dosimetry planning, and follow-up (FU) were according to the standard of care. FU data were collected through January 2025.

### Imaging and clinical data collection

Comprehensive imaging was performed prior to TARE to assess tumor burden, vascular anatomy, and treatment feasibility. Total abdominal scans were acquired on multidetector CT scanners (Siemens Somatom Definition flash Syngo (Siemens Healthineers) or Philips Brilliance 64 (Philips Medical Systems) or Spectral CT 7500 (Philips Healthcare). The contrast-enhanced CT (ceCT) acquisition protocol included a basal- and a triple-phase scan to evaluate focal liver lesions. Follow up evaluation for this study was performed on ceCT. Lesion size, localization, and vascular alterations were collected from baseline scans. Baseline clinical data collected as per standard of care for this therapeutic protocol, were used and included baseline demographic, sex, age, Barcelona Clinical Liver Cancer (BCLC) stage, albumin–bilirubin (ALBI) grade, etiology, and serum alpha-fetoprotein (AFP). Treatment-related data were collected for each patient.

### TARE procedure and dosimetry

All patients underwent a planning angiography 1–3 weeks prior to TARE, in which Technetium-99m macroaggregated albumin (^99^mTc-MAA) was injected via the hepatic artery supplying the target tumors. ^99^mTc-MAA distribution was assessed by planar scintigraphy and single photon emission computed tomography/computed tomography (SPECT/CT) on a hybrid dual-head SPECT/CT system (Discovery 670 NM/CT, GE HealthCare) to evaluate the lesion, its vascularization (feeding arteries), the perfused parenchyma, gastrointestinal shunt, and to calculate lung shunt fraction.

Inter-reader variability in contouring lesions was minimized by consensus reading as described in our previous article [7] by the first co-authors using MIM software in both centers. The tumor to normal tissue uptake ratio (TNR) was calculated to estimate tumor vascularization relative to adjacent liver parenchyma. A multicompartmental partition model was used to calculate the personalized 90Y activity to be administered with the aim of achieving a tumor absorbed dose > 120 Gy (gray) while ensuring an overall normal liver dose < 40 Gy and a lung dose < 30 Gy according to established guidelines [9, 22]. Cone Beam CT (CBCT- Philips Health Care Medical System Azurion 7 C20 or Allura) was used intra-procedurally to confirm feeding arteries to the lesions and the perfused parenchyma. Automated image reconstruction was visible after completion of each scan. The normal liver was determined on SPECT 99mTc-MAA images, while lesions were delineated on conventional cross-sectional images and correlated with 99mTc-MAA images as recommended in the guidelines [22, 23] and literature indications as the most reliable predictor of 90Y absorbed dose distribution [24, 25]. A detailed description of the two-step TARE procedure is available in a previously published paper [7]. Administered activity and targeted liver segments were individualized based on tumor characteristics and liver function. Post-TARE 90Y-PET (positive emission tomography)/CT (Discovery PET/CT 690, GE Healthcare) was used to calculate the mean absorbed tumor dose. MIM software was employed in both centers for pre-treatment and post -treatment dosimetry calculations. The multi-compartment partition model was used for pre-treatment, while voxel-based dosimetry relying on local energy deposition was applied to Y-90 PET images.

Correlation between *D*mean and objective response of the target lesion was evaluated on the 3-month follow-up imaging; correlation between extrapolated *D*mean for CR at three months was used to describe response over time.

### Assessment of efficacy and safety

Standard of care assessment of response was carried out on CT imaging at one-, three-, and six-month post-TARE and then every six months to the last time point for data collection (end of January 2025). Tumor response was evaluated using mRECIST criteria [26]; overall response (OR) rate on target lesion (ORR) was classified as complete, partial, stable, or progressive disease. Responding lesions were those with complete (CR) and partial response (PR); non-responders were defined as those with stable disease (SD) or disease progression (PD). CR was also evaluated separately. Time to local progression (TLP) on the treated lesion, overall TTP, and OS were calculated from delivery of TARE to the date of progression or to the final event from any cause. Previous, concomitant (defined within three months from TARE) and post-TARE treatments were recorded to evaluate their contribution to overall patient survival. Intraprocedural and peri-procedural adverse events were collected and classified according to the CIRSE Classification system of adverse events [27].

### Statistical analysis

Statistical analyses were performed using SPSS (v 26.0, IBM), with data visualization in GraphPad Prism (v 10.1.1). Categorical variables were reported as absolute numbers and percentages (%) and compared using the Pearson χ² test or Fisher’s exact test, as appropriate. Continuous variables were tested for normality using the Shapiro–Wilk test and expressed as mean ± standard deviation (SD) or median and interquartile range (IQR, 95% CI), as appropriate. Comparisons were evaluated using the Student’s *t*-test, or the Mann–Whitney *U*-test.

A receiver operating characteristic (ROC) curve was used to determine the optimal *D*mean cutoff for predicting the ORR on the target lesion at 3 months post-procedure. Progression and OS were estimated using the Kaplan–Meier method and compared using the log-rank test; Cox proportional hazards regression analysis was used to calculate hazard ratios (HR) with 95% confidence intervals (95% CIs) for univariable and multivariable analyses. For all analyses, a *p*-value < 0.05 was considered statistically significant.

## Results

### Patient characteristics

**Fig. 1:**
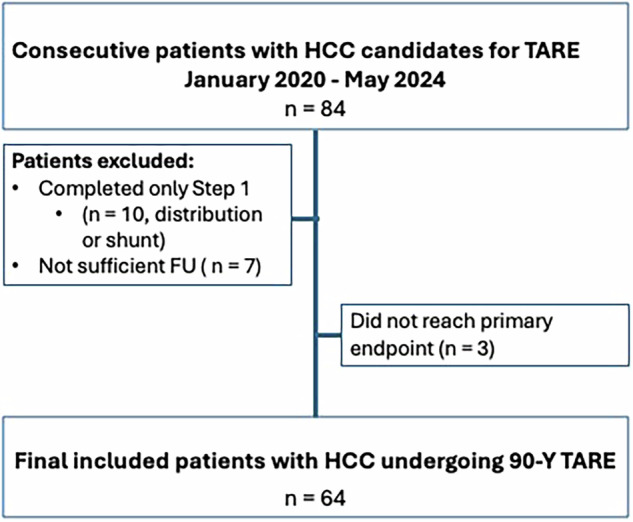
Flowchart for patient inclusion. TARE, transarterial embolization; HCC, hepatocellular carcinoma

This study included a total of 64 patients and 76 lesions from two centers (Center 1, IRCCS San Raffaele, *n* = 39; Center 2, IRCCS AOU Bologna *n* = 25, Flow chart in Fig. 1). The mean age of the cohort was 71.3 ± 9.6 years, and 54 patients (84.4%) were male. Fourteen patients (21.9%) were BCLC stage A, 26 (40.6%) stage B, and 24 (37.5%) stage C; 31 (48.4%) patients had preserved liver function with ALBI grade 1, and 33 (51.6%) had ALBI grade 2. Etiology was viral in 28 patients (43.8%); serum AFP was elevated in 42 patients (65.6%).

Twenty-five patients (39.1%) had previous treatments, which included systemic or local treatments alone or in combination, and one patient had received a liver transplant. Details are included in the Supplementary Materials. Bilobar disease was present in 22 subjects (2 patients had two treated lesions in the same lobe). In terms of multiple lesions treated, eight subjects had two lesions, and two subjects had three lesions. The mean maximum target lesion diameter on arterial phase imaging was 55.2 ± 31.8 mm. The demographic, clinical, and lesion characteristics are detailed in Table 1. Twenty-five patients (39.1%) required multiple injections due to multiple feeders (Table 2).

**Table 1:** Baseline demographic and clinical characteristics of patients (*n* = 64) and lesions (*n* = 76)

Patients	*n* = 64
Age (mean ± SD)	71.3 ± 9.6
Sex	
Male	54 (84.4%)
Female	10 (15.6%)
Etiology	
Viral	28 (43.8%)
Metabolic	19 (29.7%)
Alcoholic	4 (6.2%)
Mixed	9 (14.1%)
Other	4 (6.2%)
BCLC	
Stage A	14 (21.9%)
Stage B	26 (40.6%)
Stage C	24 (37.5%)
Previous therapy	25 (39.1%)
Concomitant systemic therapy	13 (20.3%)
ALBI grade	
1	31 (48.4%)
2	33 (51.6%)
3	0 (0%)
α-fetoprotein	
Elevated	42 (65.6%)
Normal	20 (31.3%)
Missing	2 (3.1%)
Bilobar disease	22 (34.4%)
**Lesions**	***n*** = **76**
Baseline tumor size (mm); mean ± SD	55.2 ± 31.8
Infiltrative morphology	30 (39.5%)
Necrosis	44 (57.9%)
Portal venous invasion	32 (42.1%)

Categorical variable as counts and percentages; continuous variables are expressed as mean ± standard deviations (SD)

*BCLC* Barcelona Clinic Liver Cancer, *ALBI* Albumin–Bilirubin

**Table 2:** Treatment-related parameters and complications

Patients	*n* = 64
*D*mean [Gy]; median (IQR)	261.7 (188.7–341.0)
Planned activity [GBq]; median (IQR)	1.2 (1.0–1.7)
Number of injection sites	
1	39 (60.9%)
2	22 (34.4%)
3	3 (4.7%)
Complications	
Grade 1	1 (1.6%)
Grade 2	7 (10.9%)
Grade 3	2 (3.1%)
Grade 4	0 (0%)
Grade 5	0 (0%)
Grade 6	0 (0%)

### Safety

No severe complications (Grades 4, 5, 6) were recorded. Ten (15.6%) patients had Grade 1–3 adverse events (Table 2): one patient had a Grade 1 complication (desaturation) during the procedure; seven patients (10.9%) experienced Grade 2 complications (post-embolization syndrome—fever and nausea (4), anemia, difficulty in urination, abdominal pain one week post treatment) and two patients (3.1%) Grade 3 (choledochal lithiasis; cholecystitis).

### Dosimetry

**Fig. 2:**
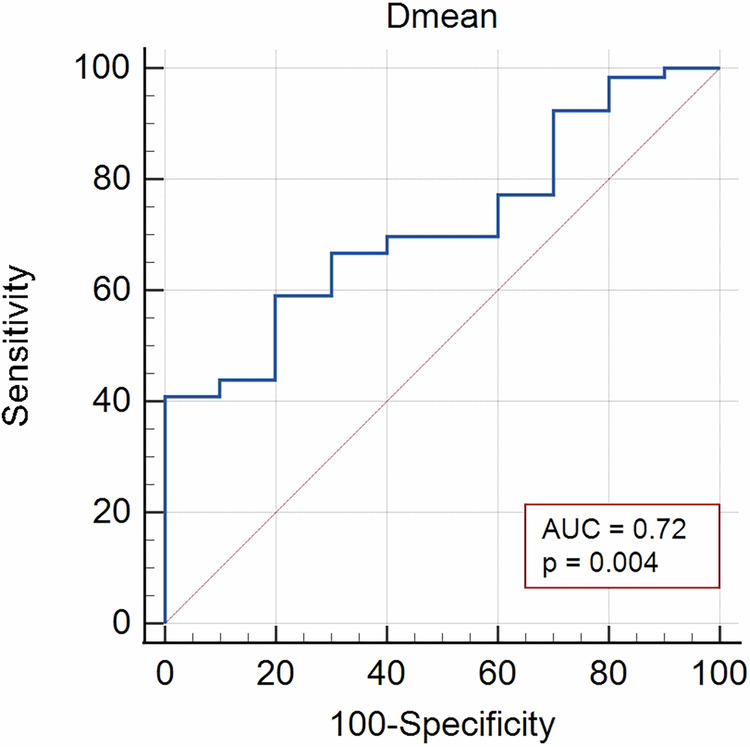
ROC curve for imean to the target lesion in predicting 3-month ORR (AUC 0.72, 95% CI 0.60–0.82; *p* = 0.004)

The median prescribed activity was 1.2 GBq (IQR 1.0–1.7) (Table 2). The median average absorbed tumor dose was used to calculate the median tumor absorbed dose of 261.7 Gy (IQR 188.7–341.0). ROC curve analysis demonstrated that a higher *D*mean to the target lesion was significantly associated with 3-month ORR (AUC 0.72, 95% CI 0.60–0.82; *p* = 0.004; Fig. 2). A *D*mean cut-off of 296.74 Gy, selected using Youden’s index, predicted ORR at 3 months with 40.9% sensitivity and 100% specificity.

We also identified a dose > 435.11 Gy, using Youden’s index (specificity 100%, sensitivity 33.3%), that predicts CR on a lesion basis in 47 lesions (= 40 patients) at three months. Those with CR at three months had CR also at 6- and 12 months (Fig. 5). ROC analysis also confirmed an association between Dmean and CR on the treated lesion at 3 months (AUC 0.63, 95% CI 0.49–0.72; *p* = 0.04; Supplementary Fig. 3).

### Outcomes

**Fig. 3:**
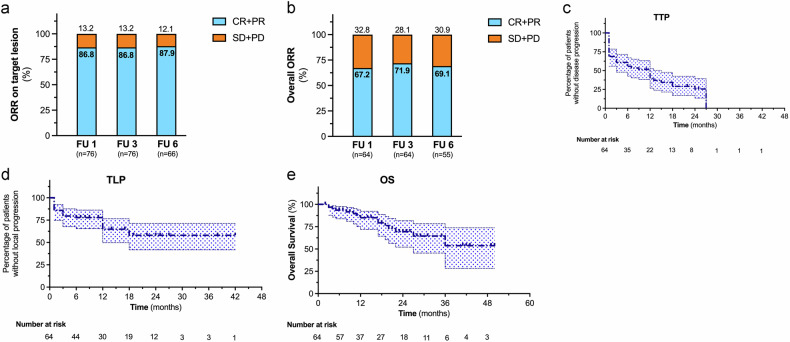
Treatment response and survival outcomes in patients with HCC treated with ^90^Y-Radioembolization. ORR for target lesion (**a**) or overall disease (**b**)—the blue bars represent the percentage of responders (CR + PR); the orange bars represent the non-responders (SD + PD). Kaplan–Meier curves showing TTP (**c**) for overall disease, TLP (**d**) for the treated lesion, and OS (**e**). CR, complete response; PR, partial response; SD, stable disease; PD, progressive disease

At 3 months, objective response on target lesion according to mRECIST showed 66 responding lesions (CR 42 + PR 24) and 10 non-responders (SD 6 + PD 4) (Table 3 and Fig. 3a). OR per patient is depicted in Fig. 3b (CR = 26; PR = 20; SD = 7; PD = 11).

**Table 3:** Follow-up (FU) outcomes and treatment response

Patients	*n* = 64
Median FU (IQR) [months]	15.0 (8.0–24.3)
Post-TARE treatments	31 (48.4%)
Surgery > 3 months	4 (12.9%)
Systemic therapy	11 (35.5%)
TACE/TAE	7 (22.6%)
Ablation	6 (19.4%)
Transplant	3 (9.7%)
Overall death	15 (23.4%)
Median time [months; (IQR)]	12.0 (6.5 - 19.5)
Cancer-related death	4 (26.7%)
Other	7 (46.6%)
Unknown	4 (26.7%)
**ORR on target**	***n*** = **76**
1-month FU	
CR	28 (36.8%)
PR	38 (50.0%)
SD	8 (10.5%)
PD	2 (2.6%)
3-months FU	
CR	42 (55.3%)
PR	24 (31.6%)
SD	6 (7.9%)
PD	4 (5.3%)
6-month FU (*n* = 66)	
CR	42 (55.3%)
PR	16 (21.1%)
SD	3 (3.9%)
PD	5 (6.6%)
**Overall ORR**	***n*** = **64**
1-month FU	
CR	16 (25.0%)
PR	27 (42.2%)
SD	10 (15.6%)
PD	11 (17.2%)
3-months FU	
CR	26 (40.6%)
PR	20 (31.3%)
SD	7 (10.9%)
PD	11 (17.2%)
6-month FU (*n* = 55)	
CR	25 (39.1%)
PR	13 (20.3%)
SD	4 (6.2%)
PD	13 (20.3%)

ORR was assessed on both target lesions and overall disease, according to mRECIST criteria. Categorical variables presented as counts and percentages; continuous variables as medians with ranges or mean ± standard deviations (SD), as specified

*CR* complete response, *PR* partial response, *SD* stable disease, *PD* progressive disease

Overall liver TTP occurred at a median time of 12.0 months (IQR 7.0–17.0 months; Fig. 3c). Median OS and median TLP were not reached; median FU time was 15.0 months (IQR 8.0–24.3). The percentage of patients without local disease progression and OS are depicted in Fig. 3d, e, respectively.

Response to target lesions in patients with different BCLC classes was not statistically significant at 1-, 3-, and 6-months (see Supplementary material). ORR in the entire liver at three months differed significantly across BCLC classes (*p* = 0.02), but not at 6 months post-TARE. The log-rank test comparing BCLC stages for overall progression was significantly different in the three classes (*p* = 0.02) (see Supplementary material).

Tumors with CR at 3-months showed a statistically significantly higher median tumor dose than those with other responses (287.6 (IQR 191.6–476.3) vs 244.9 (186.2–288.1) *p* < 0.05). Tumor diameter was not correlated to achieving CR at 3-months (*p* = 0.10); *D*mean and diameter were not correlated (*p* = 0.26, data not tabulated).

Using a dose threshold of 122.65 Gy, selected on the ROC curve as a cut-off closer to guidelines recommendations [9], patients were stratified into two groups. With this cut-off sensitivity was 92.4% and the specificity was 30.0%. Six patients (9 lesions) received doses < 122.65 Gy and 58 patients (67 lesions) > 122.65 Gy). No significant differences in TLP, TTP, or OS were observed for this threshold (see Supplementary material).

**Fig. 4:**
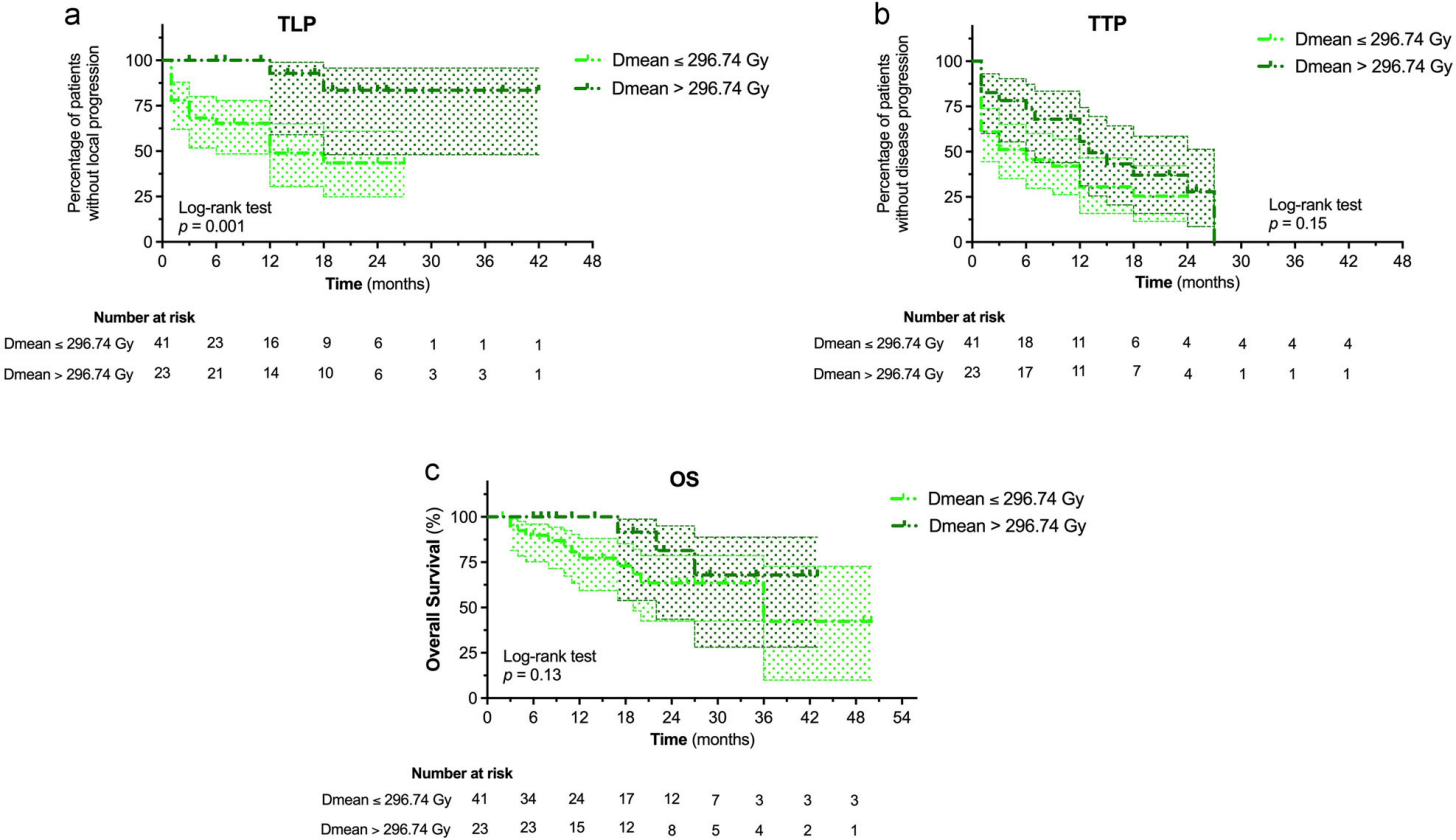
Treatment outcomes stratified by the *D*mean cut-off value of 296.74 Gy. ROC curve showing the sensitivity and specificity for the *D*mean values. Kaplan–Meier survival curves showing TLP for the treated lesion (**a**), TTP for overall disease (**b**), and OS (**c**) for patients stratified by the *D*mean. *D*mean calculated with ROC analysis; statistical significance determined using the log-rank test

The threshold identified in dosimetry as greater than 296.74 Gy, selected using Youden’s index, was used to evaluate outcomes. TLP stratified by the calculated cut-off threshold *D*mean showed a statistical difference when comparing *D*mean ≤ 296.74 to *D*mean > 296.74 Gy (log-rank *p* = 0.001) (Fig. 4a), whilst stratified overall disease progression and OS showed no significant difference (*p* = 0.15 and *p* = 0.13, Fig. 4b, c, respectively).

**Fig. 5:**
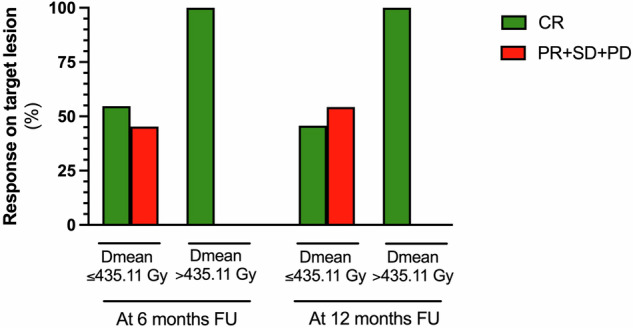
CR (green bar) vs other responses (partial response, stable disease, and progressive disease, red bar) stratified by *D*mean > 435.11 Gy at 6-months post-TARE and at 12 months FU. ROC analysis (AUC 0.63; 95% CI 0.49–0.72; *p* = 0.04) was used to determine the cut-off of CR at 3 months

Higher *D*mean to the target lesion was significantly associated with 3-month ORR (AUC 0.72, 95% CI 0.60–0.82; *p* = 0.004; Fig. 2). Doses above the threshold predicted ORR at 3 months with 40.9% sensitivity and 100% specificity. In addition, based on achieving CR on the target lesion vs all other responses at 3-months was extrapolated using a ROC curve; Youden’s index identified a *D*mean cut-off > 435.11 Gy that also predicts CR at 6-months and 12 months post TARE (Fig. 5).

Univariate analysis showed that *D*mean was the only statistically significant factor influencing TLP (*D*mean ≤ 296.74 Gy, HR = 6.9; *p* = 0.009) (Table 4). BCLC stage C and portal invasion were associated with worse prognosis with higher risk of mortality (respectively, HR = 6.0; *p* = 0.003 and HR = 3.5; *p* = 0.04) (Table 5).

**Table 4:** Univariate and multivariate analysis of factors associated with TLP

	Univariate analysis		Multivariate analysis	
	Hazard ratio (95% CI)	*p*-value	Hazard ratio (95% CI)	*p*-value
Previous therapy	2.1 (0.9–4.9)	0.10	3.6 (1.1–12.3)	**0.04**
Viral etiology	0.88 (0.4–2.1)	0.77	1.0 (0.3–3.7)	0.95
Metabolic etiology	1.2 (0.5–3.0)	0.67	0.7 (0.2–3.3)	0.67
BCLC (C)	1.5 (0.6–3.6)	0.36	6.3 (0.5–81.5)	0.16
AFP (> 7 ng/mL)	1.7 (0.6–4.6)	0.33	3.2 (0.8–12.3)	0.09
ALBI grade (grade 2)	1.8 (0.7–4.3)	0.20	2.2 (0.8–6.3)	0.15
Diameter (> 50 mm)	1.6 (0.6–3.8)	0.33	1.0 (0.3–3.6)	0.99
Necrosis	1.6 (0.6–4.0)	0.38	2.0 (0.6–6.2)	0.24
Portal venous invasion	1.0 (0.4–2.4)	0.95	0.09 (0.005–1.5)	0.09
*D*mean (≤ 296.74 Gy)	6.9 (1.6–29.9)	**0.009**	6.5 (1.3–32.5)	**0.03**
Post TARE therapy	1.6 (0.6–3.8)	0.33	1.2 (0.4–3.6)	0.71
Concomitant systemic therapy	1.2 (0.4–3.4)	0.68	1.0 (0.2–3.7)	0.99

Model fit: *x*^2^ (12) = 16.4, *p* = 0.03 (likelihood ratio test). The multivariate Cox-regression model was used for the chi-squared distribution

**Table 5:** Univariate and multivariate analysis of factors associated with OS

	Univariate analysis		Multivariate analysis	
	Hazard ratio (95% CI)	*p*-value	Hazard ratio (95% CI)	*p*-value
Previous therapy	1.5 (0.6–4.2)	0.42	1.8 (0.5–6.0)	0.37
Viral etiology	2.3 (0.8–6.7)	0.13	16.6 (1.7–164.9)	**0.02**
Metabolic etiology	0.9 (0.3–2.7)	0.82	8.6 (0.7–108.3)	0.09
BCLC stage (stage C)	6.0 (1.9–19.6)	**0.003**	9.4 (1.2–76.6)	**0.04**
AFP (> 7 ng/mL)	1.4 (0.4–4.2)	0.59	0.4 (0.1–1.8)	0.20
ALBI grade (grade 2)	2.4 (0.8–7.0)	0.13	2.2 (0.5–9.3)	0.28
Diameter (> 50 mm)	1.4 (0.5–3.9)	0.51	2.4 (0.6–10.7)	0.24
Necrosis	0.7 (0.3–2.0)	0.50	0.3 (0.1–1.1)	0.07
Portal venous invasion	3.5 (1.1–11.1)	**0.04**	1.1 (0.1–11.6)	0.93
*D*mean (≤ 296.74 Gy)	2.6 (0.7–9.2)	0.14	2.0 (0.4–9.5)	0.39
Post TARE therapy	0.9 (0.3–2.4)	0.76	1.1 (0.3–4.0)	0.87
Concomitant systemic therapy	0.7 (0.2–3.0)	0.61	0.17 (0.02–1.5)	0.11

Model fit: *x*^2^ (12) = 23.5, *p* = 0.02 (likelihood ratio test). The multivariate Cox-regression model was used for the chi-squared distribution

Multivariate analysis confirmed *D*mean as an independent predictor of progression on the target lesion (*D*mean ≤ 296.74 Gy, HR 6.5, *p* = 0.03) (Table 4) and showed that OS was independently associated with viral etiology (HR = 16.6, *p* = 0.02) and BCLC stage C (HR = 9.4, *p* = 0.04) (Table 5).

The presence of portal vein invasion did not significantly influence local progression (HR = 1.0, *p* = 0.95 univariate; HR = 0.09 and *p* = 0.09 multivariate) or occurrence of complications (*p* = 0.08, Fisher’s exact test (data not tabulated). Presence of more than one lesion or bilobar disease was not correlated to TLP (*p* = 0.33, *p* = 0.85, respectively), to overall progression (*p* = 0.21 and *p* = 0.62, respectively), nor to OS (*p* = 0.07 and *p* = 0.84, respectively) (data not tabulated).

TLP and OS were not influenced by concomitant therapy (within 3 months), (respectively *p* = 0.68; *p* = 0.61 in univariate analysis; *p* = 0.99 *p* = 0.11 in multivariate analysis). TLP was negatively influenced by previous therapy in multivariate analysis alone (HR = 3.6, *p* = 0.04). Data is available in Tables 4 and 5.

## Discussion

The partition model is the recommended approach for personalized dosimetry in the prescription and delivery of ^90^Y TARE therapy for HCC. The objective is to achieve a mean absorbed dose to the non-tumoral liver of less than 40 Gy, as well as a minimum mean target-absorbed dose of 100 to 120 Gy [9]. Elevated doses of ^90^Y-resin microspheres have been associated with enhanced tumor response and improved survival outcomes [6, 7, 13, 18, 28, 29], particularly when guided by individualized dosimetry, which ensures patient safety through meticulous dosimetric planning that minimizes radiation exposure to non-tumoral tissue. As a result, clinical guidelines are progressively favoring personalized dosing strategies over fixed dose approaches. In the literature, extensive reporting is present on personalized dosimetry in glass particles [2, 6], while still limited data is available for resin spheres.

In our cohort, the threshold calculated dose (*D*mean) was 296.74 Gy, very close to the response threshold previously reported [7]. In patients treated with doses > 296.74 Gy, the ORR (CR + PR) on the target lesion at three months was 100% compared to 79.6% for those below this threshold, regardless of BCLC stage. Dosimetry, therefore, had a significant impact on tumor progression in patients receiving doses less than or equal to the calculated *D*mean (*p* = 0.001, HR = 6.9), despite not influencing OS, most likely due to overall status and subsequent treatment options after TARE. This *D*mean dose should also be considered with respect to the diameter of the lesions (55.2 ± 31.8 m) treated, which are larger than what is reported in literature. Resin-based radioembolization segmentectomy reported by Villalobos et al [29] identified mean tumor dose thresholds associated with objective and CR at 176 Gy and 247 Gy, respectively. In a similar study [30], patients receiving a tumor-absorbed dose of ≥300 Gy had a lower disease progression rate, and a tumor absorbed dose cutoff of > 272 Gy was predictive of CR (54% sensitivity and 87% specificity). Sarwar et al [30, 31]described the correlation of hepatic tissue histology in their resin-based cohort’s post-TARE at surgery or transplant; lesions receiving a D50 of 433 Gy or greater had a statistically significant pathological complete necrosis without serious safety issues. This dose is similar to our extrapolated dose, > 435.211 Gy, for CR on imaging evaluation (33.3% sensitivity and 100% specificity) on a per-lesion basis according to mRECIST. In the cited studies, response was evaluated initially at 3 months and then at six-months, deemed as a longer period to allow the release of radiation [30–32]. The extrapolated mean dose in our cohort, closer to the guideline threshold (122.65 Gy), did not significantly influence TLP, overall progression, or OS.

Some patients receiving doses higher than 296.74 Gy and less than 435.211 Gy achieved only a partial response, which may be linked to tumor vascularization and particle number adjustment suggested by Maxwell [33]. Biodistribution is influenced by the tumor’s blood supply and heterogeneous perfusion patterns, which may require a higher particle count to ensure complete lesion coverage. The tumor-to-normal ratio may aid in identifying hypovascular areas that need to receive the same or additional dose and further optimize therapeutic outcome. Integrating CBCT volumetric segmentation [7] could enhance the prediction of final distribution volume and feeder contributions, justifying delivery of higher tumor doses.

Although the best radiological response after TARE may sometimes occur after 3 months, ORR evaluation remains a key endpoint for therapeutic decision making, which requires assessment at well-defined time points to ensure comparability across cohorts [1–3, 6, 16, 19–21]. Radiological assessments at later timepoints (e.g., 6 months) may be altered by early intrahepatic recurrence or disease progression, obscuring the true local treatment-related response and delaying the adoption of additional therapeutic options [1, 4–6].

In our cohort, no severe complications were recorded, even though the dose was more than twice the minimum guideline value. In addition, 2 patients treated with concomitant immuno-chemotherapy underwent transplants at 14- and 18- months post TARE indicating a good outcome of the combined procedure, which needs to be further investigated in a more homogenous cohort. These results, evaluated retrospectively from prospective registries, also suggest the need for more literature from larger prospective and potentially randomized cohorts, which may be supportive of higher optimized and personalized dosing regimens without severe safety concerns and good efficacy.

The calculated cut-off dose with 100% specificity and 100% PPV and over which the majority of patients responded (> 296.74 Gy), did not lead to treatment-related hepatic failure, and the two patients with grade 3 biliary injuries, who received, respectively, 272 Gy and 502 Gy, had prolonged hospital stay without severe morbidity. Bilobar disease, present in 34.4% of patients, was not associated with poorer local control, overall progression, or OS, suggesting a potential treatment option in this disease stage. The presence of BCLC class C in disease, in which portal invasion may be present, was a statistically significant factor for OS in our univariate analysis (*p* = 0.003 for stage C and *p* = 0.04 for portal vein invasion) with a six- fold hazard ratio, confirmed by the multivariate analysis stage C correlation in our cohort. It may be plausible to consider that certain descriptors of disease extent may influence the OS, even though the local tumor control (TTP and TLP) is more than adequate. One of the next steps to further characterize local outcomes would be to correlate the identified threshold *D*mean with particle number administered and with radiological CR [32, 33].

Even though this evaluation included patients from two centers with documented expertise in transarterial embolization, standard of care patient pathways and multidisciplinary approaches may slightly differ, and this may have influenced treatment indication. Post- treatment dose calculations, though, were determined with the same hardware and software. Additionally, patients enrolled had different BCLC stages representing very-early to advanced stage disease, and even though they were quite equally distributed, the number per stage was limited. The heterogeneous population may be considered a potential limitation of the study, but it reflects real-world clinical practice in high-volume centers. Prospective validation is required before adopting these dose thresholds as routine targets.

In conclusion, personalized dosimetry in ^90^Y-resin TARE for HCC has shown in this cohort to enhance tumor response rates and prolonged TTP at the identified *D*mean threshold of 296.74 Gy. Moreover, the calculated dose of greater than 435.11 Gy was associated with a maintained CR for up to one year. These findings indicated that higher absorbed doses do not appear correlated with an increase in treatment-related complications, thereby suggesting that ad-hoc dosimetry can enhance therapeutic efficacy without compromising patient safety.

## Supplementary information

Supplementary information

## Abbreviations

^90^ Y: Yttrium-90
^99m^Tc-MAA: Technetium-99m macroaggregated albumin
AFP: Alpha-fetoprotein
ALBI: Albumin–bilirubin
BCLC: Barcelona Clinic Liver Cancer
ceCT: Contrast-enhanced CT
CR: Complete response
CT: Computed tomography
FU: Follow-up
Gy: Gray
HCC: Hepatocellular carcinoma
mRECIST: Modified response evaluation criteria in solid tumors
OR: Overall response
ORR: Objective response rate
PET: Positive emission tomography
SPECT/CT: Single-photon emission computed tomography/computed tomography
TARE: Transarterial radioembolization
TLP: Time-to-local progression
TTP: Time-to-progression
