# Supplementary material for: Personalized dosimetry enhances hepatocellular carcinoma response in Yttrium-90 resin microsphere radioembolization
Source: Eur Radiol. 2026 Apr 28;36(9):7231–41. doi: 10.1007/s00330-026-12562-z (PMC13451508; doi:10.1007/s00330-026-12562-z)
Supplement: Supplementary file 1 [file 330_2026_12562_MOESM1_ESM.pdf]

PERSONALIZED DOSIMETRY ENHANCES HEPATOCELLULAR CARCINOMA RESPONSE IN YTTRIUM-90 RESIN MICROSPHERE RADIOEMBOLIZATION

ELECTRONIC SUPPLEMENTARY MATERIAL

Patients undergoing Previous Therapy	n=25
**Surgery alone**	7 (28.0%)
On target	1 (14.3%)
**Embolization alone**	5 (20.0%)
On target	2 (40.0%)
**Ablation alone**	2 (8.0%)
On target	0 (0.0%)
**Chemotherapy alone**	1 (4.0%)
**Surgery + Embolization**	3 (12.0%)
On target	0 (0.0%)
**Surgery + Ablation**	2 (8.0%)
On target	0 (0.0%)
**SBRT + Embolization**	1 (4.0%)
On target	1 (100.0%)
**Surgery + Embolization + Chemotherapy**	1 (4.0%)
**Surgery + Embolization + Ablation**	1 (4.0%)
On target	1 (0.0%)*
**Chemotherapy + Radiotherapy on other tissue lesions°**	1 (4.0%)
**Transplant**	1 (4.0%)

Supplementary Table 1. Previous treatments before 90^{Y}-Radioembolization.

A subgroup of 25 HCC patients received prior therapies before 90^{Y}-Radioembolization. “On target” indicates cases in which the previous treatment was delivered to the same lesion subsequently treated with TARE. Percentages in the “On target” column are calculated within each treatment subgroup. *surgery was performed on target lesion; °vertebral lesions.
Abbreviations: SBRT, Stereotactic Body Radiation Therapy.

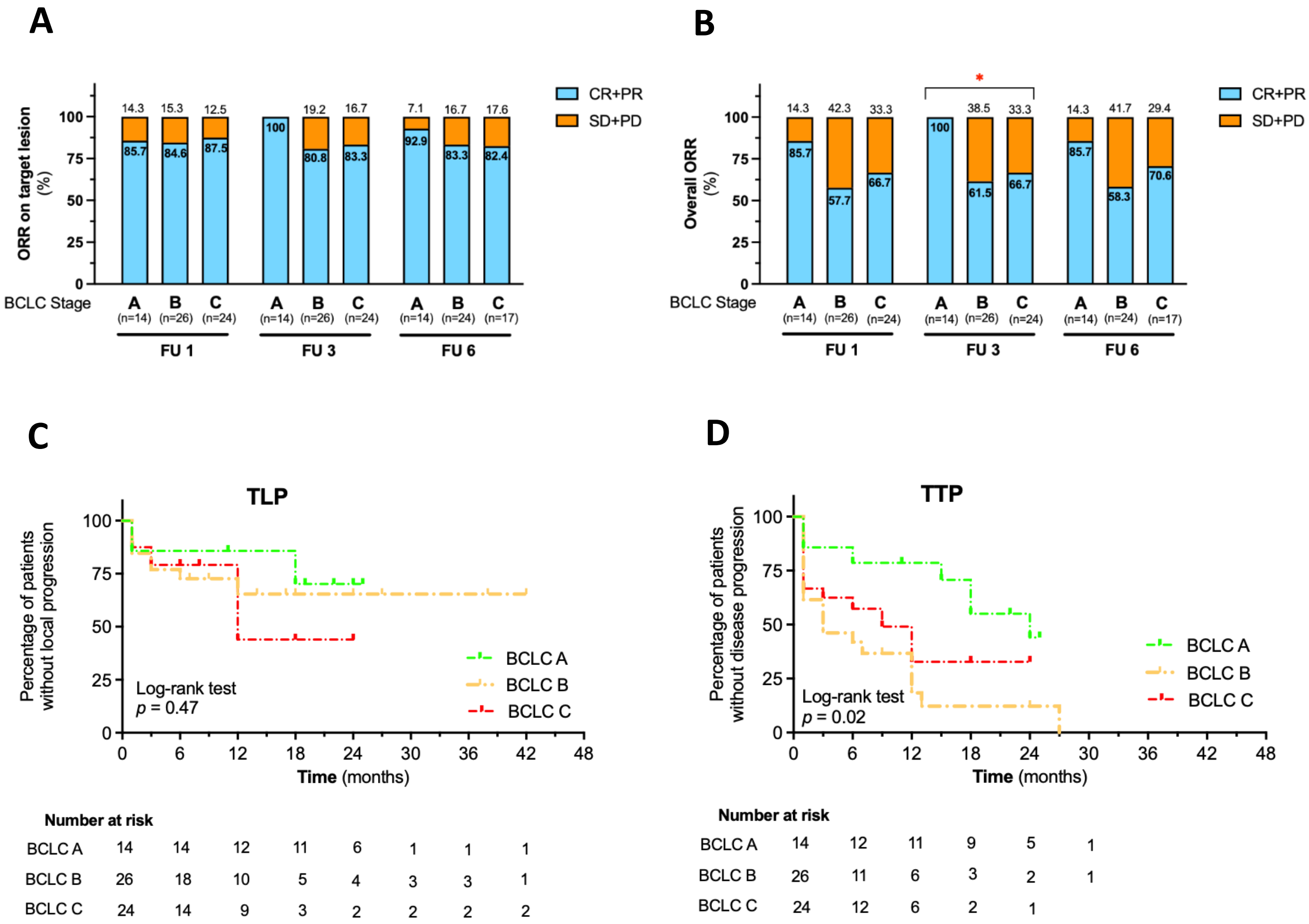

Supplementary Figure 1. Treatment outcomes stratified by BCLC stage.

(A-B) Objective response rate (ORR) for **(A)** target lesion or **(B)** overall disease stratified by BCLC Stage. The blue bars represent the percentage of responders (CR+PR), while the orange bars show the non-responders (SD+PD). Statistical comparison was performed using Fisher's exact test; *p* = 0.02.

(C-D) Kaplan-Meier survival curves showing **(C)** LTP for the treated lesion and **(D)** TTP for overall disease stratified by the BCLC Stage. Analysis performed using the log-rank test.

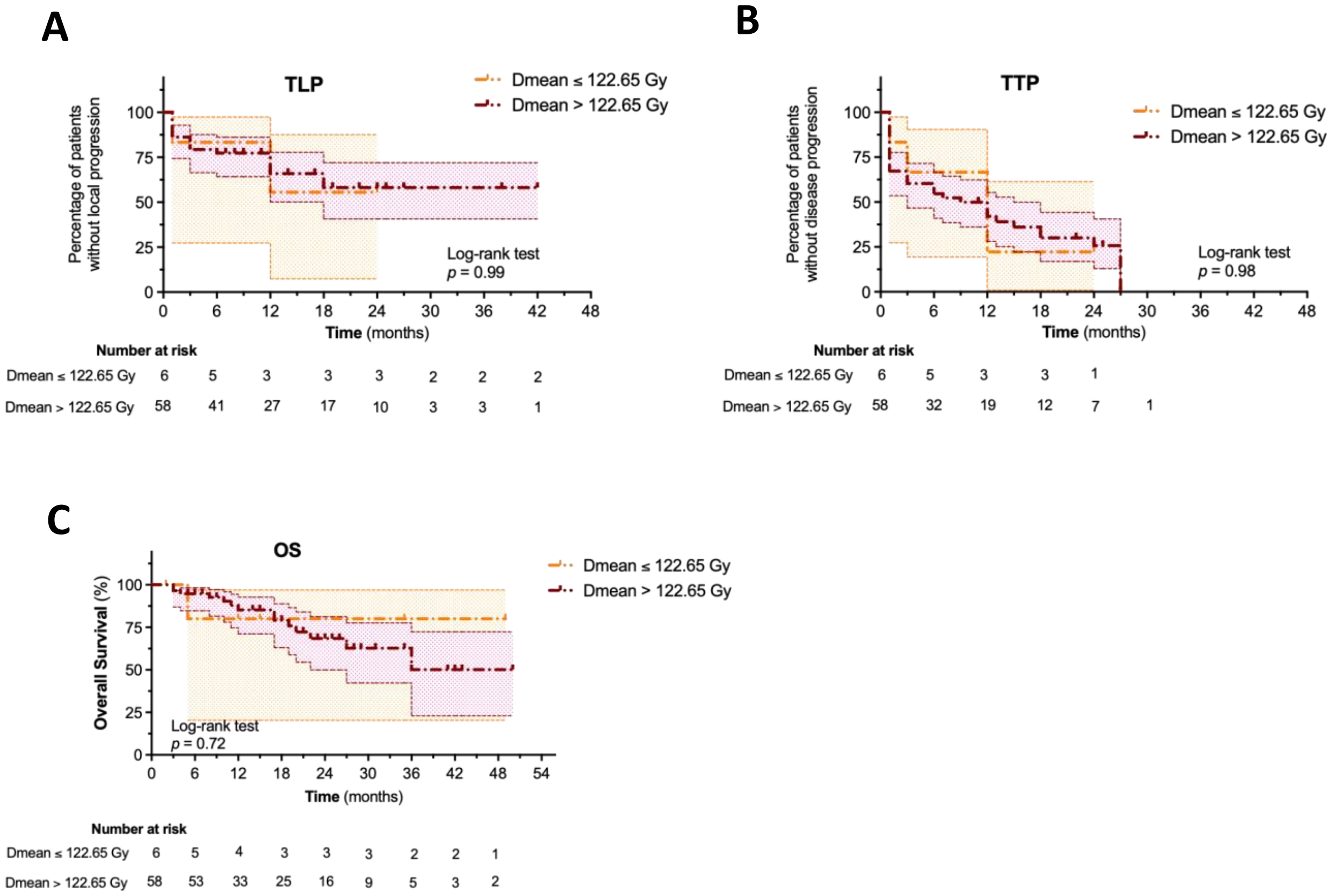

Supplementary Figure 2. Treatment outcomes stratified by Dmean value of 122.65 Gy. **(B-D)** Kaplan-Meier survival curves showing **(B)** LTP for the treated lesion, **(C)** TTP for overall disease and **(D)** OS for patients stratified by the Dmean value of 122.65 Gy. Statistical significance was determined using the log-rank test.

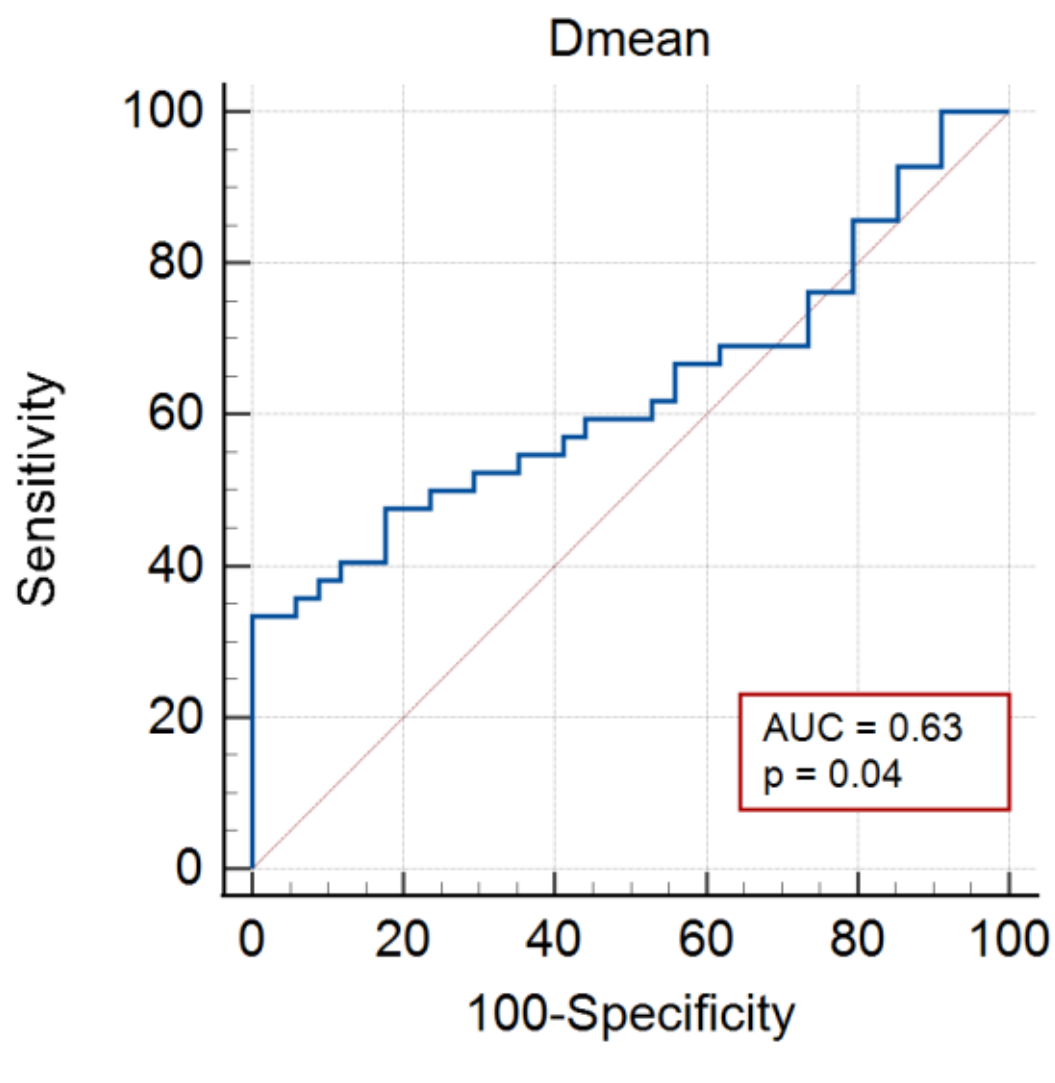

Supplementary Figure 3. ROC curve showing the sensitivity and specificity for the Dmean cut-off value at three months after TARE that predicts complete response (CR).

Eur Radiol (2026) Di Gaeta E; Taninokuchi Tomassoni M; Calabrese F et al.
